# CRE: An R package for interpretable discovery and inference of heterogeneous treatment effects

**DOI:** 10.21105/joss.05587

**Published:** 2023-12-15

**Authors:** Riccardo Cadei, Naeem Khoshnevis, Kwonsang Lee, Daniela Maria Garcia, Falco J. Bargagli Stoffi

**Affiliations:** 1 Department of Biostatistics, Harvard School of Public Health, Boston, Massachusetts, United States of America; 2 Department of Computer and Communication Science, École Polytechnique Fédérale de Lausanne, Lausanne, Switzerland; 3 Research Computing, Harvard University, Cambridge, Massachusetts, United States of America

## Abstract

In health and social sciences, it is critically important to identify interpretable subgroups of the study population where a treatment has notable heterogeneity in the causal effects with respect to the average treatment effect (ATE). Several approaches have already been proposed for heterogeneous treatment effect (HTE) discovery, either estimating first the conditional average treatment effect (CATE) and identifying heterogeneous subgroups in a second stage (Bargagli-Stoffi et al., 2020, 2022; Foster et al., 2011; Hahn et al., 2020), either estimating directly these subgroups in a direct data-driven procedure (Nagpal et al., 2020; Wang & Rudin, 2022). Many of these methodologies are decision tree-based methodologies. Tree-based approaches are based on efficient and easily implementable recursive mathematical programming (e.g., HTE maximization), they can be easily tweaked and adapted to different scenarios depending on the research question of interest, and they guarantee a high degree of interpretability—i.e., the degree to which a human can understand the cause of a decision (Lakkaraju et al., 2016). Despite these appealing features, single-tree heterogeneity discovery is characterized by two main limitations: instability in the identification of the subgroups and reduced exploration of the potential heterogeneity. To accommodate these shortcomings, Bargagli-Stoffi et al. (2023) proposed Causal Rule Ensemble, a new method for interpretable HTE characterization in terms of decision rules, via an extensive exploration of heterogeneity patterns by an ensemble-of-trees approach. CRE is an R package providing a flexible implementation of Causal Rule Ensemble. The package allows for multiple variants of Causal Rule Ensemble algorithm, also including different internal individual average treatment effect (IATE) estimators—i.e., AIPW (Robins et al., 1994), Causal Forest (Athey et al., 2019), Causal BART (Hill, 2011), S-Learner (Hill, 2011), T-Learner (Hansotia & Rukstales, 2002), X-Learner (Künzel et al., 2019).

## Statement of Need

Several methodologies for HTE estimation have already been proposed (together with the release of the corresponding packages), but the interpretable discovery of the subgroups and the key factors driving the HTE is still an open challenge. To the best of our knowledge, causalTree, based on Causal Honest Tree (Athey & Imbens, 2016), is the unique R package proposing a methodology for interpretable HTE discovery and estimation via decision rules. Still, despite its appealing features, it is also characterized by the limitations of single tree-based methods. Firstly, single-tree-based subgroup identification is sensitive to variations in the training sample (high model variance)—e.g., if the data are slightly altered, a completely different set of discovered subgroups might be found (Breiman, 1996; Hastie et al., 2009; Kuhn et al., 2013). Secondly, it may fail to explore a vast number of potential subgroups (limited subgroup exploration)—e.g., the subgroups discovered are just the ones that can be represented by a single tree (Kuhn et al., 2013; Spanbauer & Sparapani, 2021). To illustrate, consider a scenario in which two distinct factors independently contribute to the heterogeneity in treatment effects. In such cases, a single tree algorithm may detect only one of these factors, failing to identify the second. In instances where both factors are identified, they are detected sub-optimally as an interaction between the two variables rather than as distinct drivers of the treatment heterogeneity. To account for these shortcomings, we propose CRE (Khoshnevis et al., 2023), an R package providing a flexible implementation of the Causal Rule Ensemble algorithm. CRE provides (i) an interpretable representation of the HTE in observational studies, (ii) via an extensive exploration of complex heterogeneity patterns using decision rules, while (iii) guaranteeing high stability in the discovery.

## Algorithm

Causal Rule Ensemble relies on the Treatment Effect linear decomposition assumption, which characterizes the Conditional Average Treatment Effect (CATE) as the sum of M+1 distinct contributions:

$$\tau \left(x\right)=\text{E}\left[\tau_{i}|X_{i}=x\right]=\bar{\tau }+\sum_{m=1}^{M}\alpha_{m}\cdot r_{m}\left(x\right)$$

where $\bar{\tau }$ is the ATE, $\tau_{i}$ is the ITE, and for each m in $\left\{1,\ldots ,M\right\}$, $r_{m}$ is an interpretable decision rule characterizing a specific subset of the covariate space, and $\alpha_{m}$ is the corresponding Additive Average Treatment Effect (AATE). The CRE procedure is divided into two steps, discovery and inference, and each observation is used for only one of the two steps (honest splitting). The splitting is at random and the percentage allocated to each step is controlled. During the discovery step, CRE retrieves the M decision rules characterizing the heterogeneity in the treatment effect. A set of candidate decision rules is extracted by an ensemble of trees modelling some IATE estimates (Dorie et al., 2020; Polley et al., 2019; Tibshirani et al., 2023) (*fit-the-fit* approach), and among these, only a simple and robust subset of rules is selected for the linear decomposition by the Stability Selection algorithm via LASSO (Friedman et al., 2021; Hofner et al., 2015; Meinshausen & Bühlmann, 2010). During the inference step, CRE estimates the ATE and AATEs, by the normal equations to model some IATE estimates and confidence intervals are provided by bootstrapping. A brief schematic summary of the described procedure (Bargagli-Stoffi et al., 2023) is reported below.


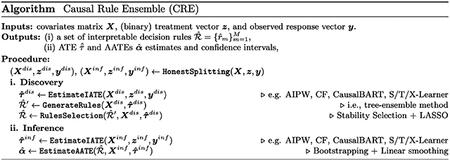


## Usage

CRE is available both on CRAN (Khoshnevis et al., 2023) and GitHub, and can be installed and loaded into the R session using:


install.packages(“CRE”) 
library(“CRE”)


generate_cre_dataset() is a flexible synthetic dataset generator, which can be used for simulations before applying CRE to real-world observational data sets. It generates an outcome array y (binary or continuous), a treatment array z (binary), a covariate matrix (binary or continuous) and the true (unobserved) individual treatment effect ite (useful for performance evaluation). The input parameters specify the dataset characteristics, including the number of individuals (n), the number of covariates (p), the correlation within the covariates (rho), the number of decision rules (n_rules) decomposing the CATE, the treatment effect magnitude (effect_size), the confounding mechanism (confounding), and whether the covariates and outcomes are binary or continuous (binary_covariates, binary_outcome). For a full description of the data generating process and its variants, see Section 4 in Bargagli-Stoffi et al. (2023).


set.seed(2023)
dataset <- generate_cre_dataset(n = 5000,
                                       rho = 0,
                                       n_rules = 4,
                                       p = 10,
                                       effect_size = 5, 
                                       binary_covariates = TRUE, 
                                       binary_outcome = FALSE, 
                                       confounding = “no”)
y <- dataset$y
z <- dataset$z
X <- dataset$X
ite <- dataset$ite


We propose here three examples of how to run the Causal Rule Ensemble algorithm by the CRE package.

### Example 1.

Running Causal Rule Ensemble with default parameters. For a detailed description of the default method and hyper parameters refer to https://nsaph-software.github.io/CRE/articles/CRE.html.


results <- cre(y, z, X)


### Example 2.

Running Causal Rule Ensemble with customized IATE estimator. If ite argument is provided, the IATE estimation both in the discovery and inference step is skipped. This argument is useful either to consider new IATE estimators which are not internally implemented in this package, either to compute this estimation una tantum (saving the results) and speed up the execution time during the hyper-parameter tuning.


# personalized IATE estimation (S-Learner with Linear Regression)
model <- lm(y ~., data = data.frame(y = y, X = X, z = z)) 
iate_pred <- predict(model, newdata = data.frame(X = X, z = z))
results <- cre(y, z, X, ite = iate_pred)


### Example 3.

Running Causal Rule Ensemble with customized parameters (no need to explicit all the arguments). The method parameters (method_params) entail the IATE estimator method, the outcome and propensity score learners, if needed), and the the ratio of data to use for each step. The hyper parameters (hyper_params) entail all the other parameters through which Causal Rule Ensemble algorithm can be fine tuned. For a detailed description of all the method and hyper parameters refer to https://nsaph-software.github.io/CRE/articles/CRE.html.


method_params <- list(ratio_dis = 0.5, 
                             ite_method = “aipw”, 
                             learner_ps = “SL.xgboost”, 
                             learner_y = “SL.xgboost”)
hyper_params <- list(intervention_vars = NULL, 
                            offset = NULL,
                            ntrees = 20,
                            node_size = 20,
                            max_rules = 100,
                            max_depth = 3,
                            t_decay = 0.025,
                            t_ext = 0.025,
                            t_corr = 1,
                            t_pvalue = 0.05, 
                            stability_selection = “vanilla”, 
                            cutoff = 0.9,
                            pfer = 0.1,
                            B = 50,
                            subsample = 0.05)
results <- cre(y, z, X, method_params, hyper_params)


The results are collected in a S3 object containing: the number of decision rules extracted at each step (M), the list of the rules finally selected (rules), a data.frame with the CATE decomposition estimates with corresponding uncertainty quantification (CATE) and the list of selected parameters (method_params and hyper_params).

summarize() and print() display a summary of these results. predict() estimates the Individual Treatment Effect on a new Covariate matrix by the linear decomposition just learnt. plot() visualizes the CATE decomposition estimates in a range bar plot.


print(results)
y_pred <- predict(results, X) 
plot(results)


**Figure 1:**
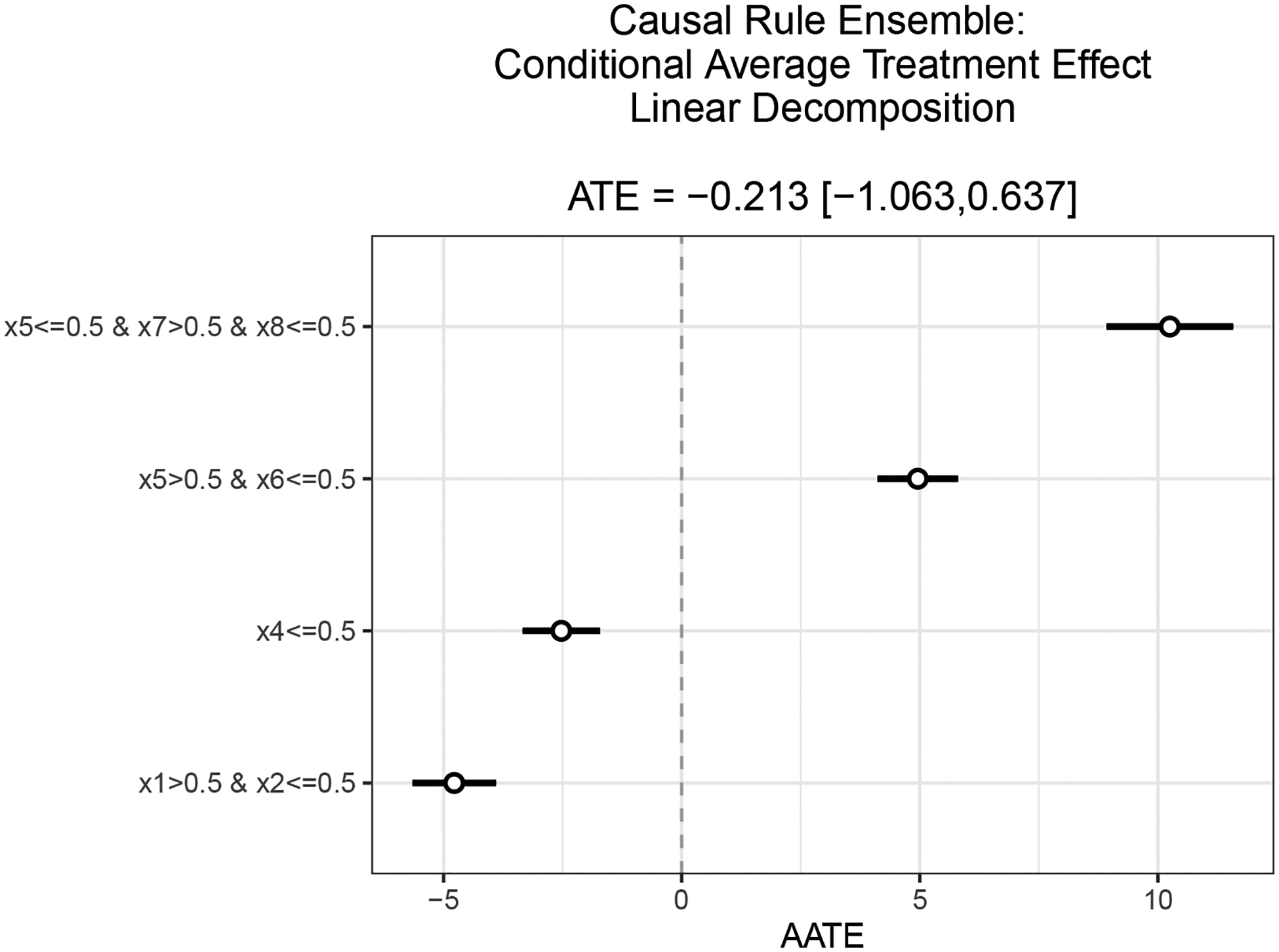
Visualization of Causal Rule Ensemble HTE linear decomposition for Example 3. For each decision rule discovered, the corresponding AATE estimate with 95% confidence interval is reported in a range bar plot. The decision rules are ordered from the most vulnerable (high AATE) to the least, and the ATE is reported on top of the plot.

Figure 1 reports the visualization of the results for Example 3, which perfectly discover the correct CATE decomposition.

**Figure 2:**
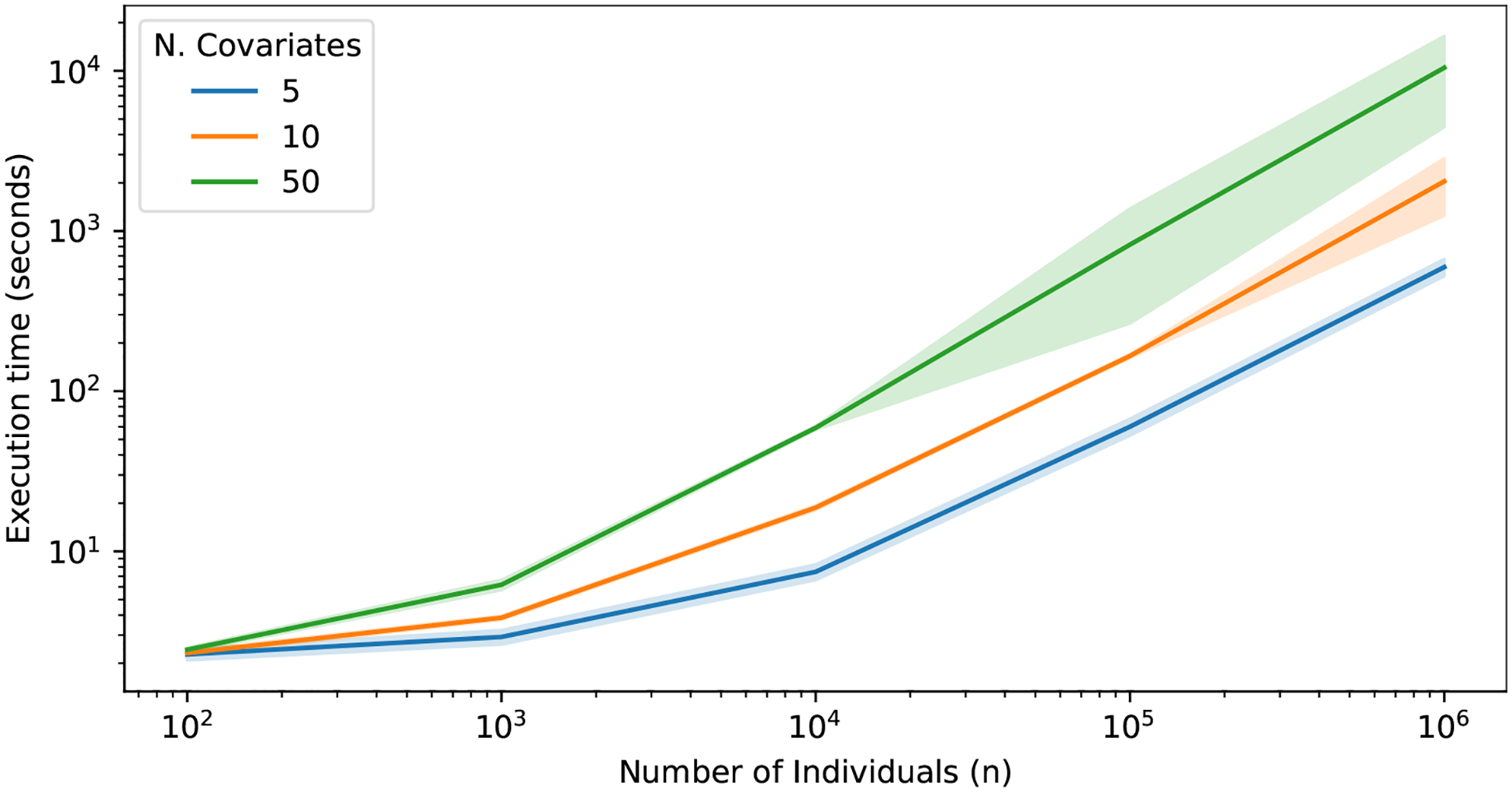
Log-Log line plot reporting the average execution time of cre() and standard deviation over 10 seeds per experiment, varying the number of individuals and observed covariates.

The observed average execution time of the method varying the number of individuals and observed covariates on R 4.2.1 running on macOS 12.6 on a MacBook Pro 16GB Apple 8-cores M1 processor is reported in Figure 2.

Online documentation for the package can be found at https://nsaph-software.github.io/CRE/.
